# A qualitative exploration of the role of NGOs in the recovery support for persons with substance use disorders in a low-income African setting

**DOI:** 10.1186/s13011-021-00400-y

**Published:** 2021-08-17

**Authors:** Kwaku Oppong Asante, Emmanuella Asiama-Sampong, Richard Appiah

**Affiliations:** 1 Department of Psychology, University of Ghana, P. O. Box LG 84, Legon Accra, Ghana; 2 Department of Psychology, University of the Free State, Bloemfontein, South Africa; 3 College of Health Sciences, University of Ghana, Accra, Ghana

**Keywords:** Substance use disorders, Recovery support services, Non-governmental organizations, Mental health, Ghana

## Abstract

**Background:**

In sub-Saharan Africa, most government mental health facilities are under-resourced to cater for the mental health needs of the population, including the provision of treatment and recovery support services for persons with substance use disorders (SUDs). However, in other settings, non-governmental organizations (NGOs) play significant roles by complementing governments’ efforts in the provision of care for vulnerable groups. Presently, no study exists that examines the contributions of NGOs in the recovery support of individuals with SUDs in the Ghanaian context. This study sets out to explore the role of NGOs in the recovery of persons with SUDs in Ghana.

**Method:**

Using a descriptive qualitative design, eight staff (directors and senior recovery practitioners) from eight NGOs in southern Ghana were purposively selected and interviewed face-to-face using semi-structured interview guide. The interviews were audio-taped, transcribed verbatim, and analyzed using the thematic analysis within an inductive approach.

**Results:**

The results showed that NGOs provide three main services: treatment of drug addiction (through psychotherapy and recovery capital), re-integration of recovered individuals into society, and advocacy and awareness creation in schools and communities. These efforts are thwarted by limited qualified professionals and inadequate government support.

**Conclusion:**

Our results underscore the need for government agencies to collaborate with NGOs involved in the recovery management of persons with SUDs and other mental disorders to complement their efforts in strategizing, designing, and implementing context-appropriate substance misuse prevention and intervention programs and policies in Ghana.

## Introduction

A wealth of evidence exists that demonstrate the adverse effects of substance misuse on the individual, family, and society [1, 2]. Substance misuse – the use psychoactive substances in a way that causes harm to the user and others – has been associated with substantial social, economic, and health problems on the individual, family, and society, including mental illness [3], motor accidents [4], increased crime [5], lost productivity [6], economic burden and cost of treatment [7], and death from overdose [8].

In most sub-Saharan African countries, mental health care, compared to biomedical care, has received little priority from governments and health planners in the last decade. For instance, in Ghana, less than 1% of the overall annual health budget was allocated to mental healthcare, over the past two decades [9]. There are only three national psychiatric hospitals in Ghana – with two located in the southern part of the country. Of note, there have been recent efforts to establish mental health units in all regional hospitals in Ghana [10, 11]. Nonetheless, these facilities are all located in urban areas with the majority solely owned by the private sector. Presently, there is a wide disparity of mental health care services in Ghana, particularly in the rural settings and northern sector of the country [12]. Although there are no recent published data on the proportion of the population with psychopathology, data from a decade old study estimated that about 1.2 million Ghanaians have alcohol and drug-related problems in 2010 [13]. A more recent data from the regional-level health facilities also suggests an increasing trend in substance misuse [14]. The same study reported that 1839 patients were treated for alcohol and drug-related (mental) health problems at the Sunyani Regional Hospital in 2015, compared to 1046 patients in 2014 and 963 patients in 2013 [14]. Confronted with a myriad of challenges, including inadequate and under-resourced health facilities as well as the lack of mental health specialists, the needs of patients and families with mental disorders, particularly individuals in treatment for substance use disorders (SUDs), far outstrip the available facilities and services provided by the government [15].

The last decade has seen the presence and emergence of several non-governmental organizations (NGOs) in the national developmental agenda of Ghana. Presently, there are about 382 registered NGOs and related organizations across all the 16 regions of Ghana [16, 17], with the majority centered in the northern sector. The majority of these NGOs, such as the Innovations for Poverty Action (a US-based non-profit specialized in intervention research using randomised controlled trials), are focused on poverty alleviation and social care among rural poor populations. Other NGOs, including BasicNeeds, Mental Health Society of Ghana, Psycho-Mental Health International, MindFreedom, and Friends of Mental Health provide mental health care services to specific target groups. The majority of NGOs in Ghana, and sub-Saharan Africa more generally, are driven by a passion to support economic-deprived groups to cultivate or access good food and water, provide formal and informal education for vulnerable children and adults, and provide free or affordable health care to vulnerable populations [18].

There is growing evidence of the critical contributions of NGOs to the national development in other settings [19–21]. In Ghana, a significant number of registered NGOs exist to promote health care, including mental health. With the reported increasing rate of substance misuse and relapse to SUDs in Ghana [14], there is need to institute a collaborative effort to tackle this public health crisis. Considering that most health-focused NGOs work with vulnerable groups, including persons with SUDs, there is need to explore and understand the roles of these organizations in the recovery management of these vulnerable groups, including the recovery capital (i.e., the sum of internal and external resources necessary to initiate and sustain recovery from substance misuse) that they offer to clients. The overarching aim of this study was to explore the role of NGOs in the recovery management of persons with SUDs in Ghana. Specifically, the study aimed to answer the following questions: 1) What activities and services do mental health-focused NGOs within the Accra metropolis provide to support persons with SUDs in Ghana? and 2) What challenges are these NGOs confronted with in their execution of these roles? Information on the contributions of NGOs in the provision of these services can inform governments, health agencies, corporate and social organizations, groups, communities, and individuals about the roles and challenges confronting these organizations, envision areas for possible collaborations, guide the formulation of context-appropriate educational and intervention programs and policies for individuals, schools, and communities.

## Methods

### Research design and setting

We implemented a qualitative approach with exploratory and descriptive design to gain full understanding of the roles of the NGOs in the treatment and recovery of persons with SUDs in Ghana. Qualitative research allows researchers to explore an in-depth understanding of participants’ behaviors, perspectives, feelings, and experiences and represents data that preserves the participants’ world [22]. The study was conducted within the Greater Accra Region (GAR) of Ghana. The GAR is the national capital of Ghana and one of the 16 administrative regions of Ghana. The region is located in the southern part of Ghana with a population of about 4 million in 2010 [23], although the smallest. The GAR has the majority of NGOs that provide mental health services to individuals in Ghana [16].

### Population and sampling

The study targeted directors and senior recovery practitioners who work with registered NGOs within the Greater Accra metropolis. The inclusion criteria were that: 1) the participant should have a managerial portfolio in an NGO that was involved in the provision of treatment and recovery support services for individuals with SUDs; 2) the participant should have worked in the NGO for a minimum of three years; 3) the NGO should have operated for a minimum of five years; and 4) the NGO should have provided mental health support or services to at least 20 persons with SUDs. Purposive sampling technique was used to recruit individuals who met the inclusion criterion and consented to participate in the study.

### Ethics and recruitment of participants

The study was approved by the Department of Psychology Research & Ethics Committee (Ethics number: DREC/001/17–18) at the University of Ghana. Ethical approval documentations were submitted to 13 randomly selected health-based NGOs in the GAR, of which eight agreed to participate. Verbal and written informed consent were obtained from all participants. All participants were assured of anonymity and the processes instituted to ensure confidentiality were thoroughly explained to participants. Participants were informed about their right to withdraw from the study at any time without any consequences. The information sheet were presented to all selected individuals who were allowed a week to finalize their decision to participate. Only individuals who consented to participate were recruited.

### Data collection and management

Data was collected through individual face-to-face in-depth interviews using a semi-structured interview guide. All interviews were conducted in English, at the office premises of each selected NGO (as suggested by participants) and audiotaped with participants’ permissions. Each interview lasted for about forty-five (45) minutes and focused on issues relating to the provision of treatment and recovery support services for persons with SUDs in the GAR. General open questions asked include: *Please describe to me the main activities (functions) of your NGO*? *What services do you provide for your target group – in terms of treatment and recovery support? What challenges do your organization face when carrying out these functions*? To avoid the researcher’s own biases, leading questions were avoided during the process of data collection. Based on participants’ responses, follow-up questions were posed for clarification and to gain full understanding of the roles of these NGOs.

### Data analysis

The principles and techniques of thematic analysis were followed to analyze the data [24]. First, transcripts were read and re-read to gain an understanding of participants’ perspectives. Subsequently, the first and second authors independently (and manually) coded, categorized, and searched for patterns and meanings relevant to the aims and objectives of the present study. In the next stage, the authors revisited and reviewed the identified themes to ensure that they represented the emerged patterns. Subsequently, the coded statements were arranged under different broad themes – which were thereafter re-examined by the authors to validate them. The two co-coders discussed each of the emerged theme to reach consensus. Codes were assigned to verbatim quotes that describes each theme.

### Trustworthiness

In other to ensure that the results of this study were valid, the steps and guidelines postulated by Creswell [22] and Lincoln and Guba [25] were followed. To ensure credibility, we adopted strategies to increase honesty in the participants. Participants were encouraged to participate in the study by volition, without any coercion. Furthermore, all participants were interviewed with the same interview guide using iterative questioning to follow-up on participants’ responses and comments and to confirm understanding derived from comments on in order to arrive at a more detailed description of their accounts. Detailed field notes were written up during data collection and analysis to ensure auditability of the study [26]. To ensure transferability and dependability, a detailed description of the study background, context, and methods has been provided to enable replication of the study. Prolonged engagement was employed to ensure that detailed accounts were obtained in this study.

## Results

### Participants’ characteristics

Six of the NGOs have been operational in Ghana for 16 years on average, have about 12 employees each, and together have provided services for about 208 individuals with various substance misuse related problems within the past year. The remaining two NGOs have operated for nine and six years, have an average of 11 employees, and together have provided services for about 94 individuals with various substance misuse and mental health related problems in the past year. The participants in the study were made up of eight adult individuals aged between 38 and 52 years. Three of the participants were directors, whereas the remaining five were senior recovery practitioners. All participants had worked with their respective NGOs within a year range of 6 to 16. Table 1 presents the descriptive profile of participants.

**Table 1:** Descriptive characteristics of participants

Participants	Age (years)	Role in the NGO	Years of working
1	50	Director	15
2	48	Director	18
3	51	Director	12
4	40	Senior recovery practitioner	10
5	50	Senior recovery practitioner	12
6	42	Senior recovery practitioner	8
7	38	Senior recovery practitioner	9
8	41	Senior recovery practitioner	7

The study revealed three themes that describe perspectives of participants on the role of their NGOs in the recovery support for persons with SUDs in Ghana. The emerged roles include the provision of psychotherapy and recovery capital, re-integration of recovered individuals into society, and advocacy and awareness creation. For the challenges confronting these health-focused NGOs in their lines of duty, the findings revealed logistical and personnel challenges, including inadequate government support and inadequate qualified professionals.

#### Provision of treatment services

One primary function of the NGOs, as revealed by the data, is their involvement in the provision of treatment and recovery support services for individuals recovering from SUDs. As a core function, health-oriented NGOs working with persons with SUDs mostly provide psychiatric and psychological services for their clients. Resident and non-resident clinicians are engaged to provide ongoing medical and psychological interventions for these clients – who are often housed in facilities by the NGOs. Two themes: psychotherapy and psychological capital were identified under this main theme.

##### Psychotherapy

The majority of individuals with mono- or poly-substance use disorders also contend with some mental health problems, either as the precipitant to the use (and misuse), or as a consequence of the disorder. With the myriad of associated psychosocial problems, individuals with SUDs often require extensive medication (psychotropics) to regulate the neurotransmitters and chemical imbalances that underpin their mental health problems. Given that a number of individuals tend to develop behavior problems and psychopathological symptoms as a result of the misuse, the provision of psychotherapy is necessitated. Psychotherapy involves the application of psychological methods to explore a person’s moods, feelings, thoughts, and behaviors and the problems associated with them, and help them to develop strengths and strategies to overcome them. The majority of NGOs have resident and visiting psychologists who provide psychotherapy sessions for individuals and groups on weekly basis. One participant, a 50-year old male director recounted:

*“…since most of our clients have serious addiction problems that urge them to continue to use the substances, we have clinical psychologists who come in to help them, using psychotherapy. They discuss their addiction problems and help them find ways to overcome it…almost all our clients are taken through this step. Occasionally, the psychologist also organizes a group session, like the Alcohol Anonymous Ghana, for those who have almost recovered, or those who have been discharged home but are required to visit weekly or every two weeks…” [Participant 1].*

*“…for those who come here, a number of them present with psychological problems and need the services of a clinical psychologist. They [psychologist] assist them to identify the challenges instigating the addictive behavior and try to find ways to help them to stop…it [psychotherapy] is the main treatment that we offer them here. A few people are sometimes taken to see the psychiatrist for assessment and medication when necessary…” [Participant 3].*

##### Recovery capital

In addition to the provision of psychological services, mental health-oriented NGOs are also concerned with assisting their clients to access other health related services and to reconnect with their families and communities. Participants narrated that one of their key roles involves advocating for their clients to re-integrate or resume their previous jobs, if possible. There is sufficient empirical evidence that demonstrate the crucial role of recovery capital in the recovery process of individuals with SUDs. Across populations and contexts, researchers and clinicians have articulated the need to institute and enhance social support systems, cultural and religious activities, access to effective healthcare and accommodation, as well as the use of recreational activities in the recovery process of people in treatment for SUDs and other related substance-induced mental health problems. A 42-year old male senior recovery practitioner stated:

*“…most of our clients do not have the support systems necessary to keep them sober or prevent them from relapsing…one of our key functions is to mobilize resources to support those who have been rehabilitated to establish connections with their families and communities or start their own businesses or advocate for their employers to accept them back to their former jobs…” [Participant 6].*

*“…we also ensure that our clients receive adequate support from their families and workplaces…some of them are assisted to live with their families when possible…due to the stigma associated with mental health problems, most families are not willing to accept these people back into the family…” [Participant 5].*

To provide continuous support and minimize the likelihood that recovered individuals relapse to substance misuse, most NGOs offer after-discharged services, where recovered clients visit the facilities once-weekly or fortnightly for review. Clinicians and staff review clients' progress by exploring their challenges and offer practical suggestions to help them to overcome plausible high risk situations. Such continuous encounters and support are important to complement clients’ efforts to ensure long-term sobriety.

In some cases, recovery capital has to do with individuals participating in weekly reviews at the recovery support centers. This is elaborated by one of the participants below:

*“…we also have arrangement with the clinical psychologist to attend to those who are no more here at the facility…about once a week or so…to evaluate their progress and offer further counseling support as and when necessary…” [Participant 5].*

##### Re-integration and skill acquisition

While mental health affiliated NGOs provide comprehensive services to support the recovery process of clients, these services are intended to be on the short- to medium-term. The services and facilities are often not designed for long-term care. Generally, individuals are housed until they are fully recovered – which can span from a couple of weeks to several months. Research evidence shows that community-based care, in general, is more effective and therapeutic compared to facility-based care. The overarching goal of most mental health affiliated NGOs is therefore to assist recovered individuals resume their pre-morbid status and to pursue a meaningful and purposeful life in their homes and communities. The NGOs run a set of skill-based programs, such as hairdressing, weaving artifacts for females and repairing (electronic devices) for males who do not have any skill. After completion of training and acquisition of the requisite skills, the NGOs provide each graduated individual with a set of working tools and startup cash prizes to start a small business. A 41-year old senior recovery practitioner mentioned:

*“…what we want to see is the person has fully recovered and functioning well in their communities…and back in their families. We also give them some skills training and support them to start their businesses – especially for those who are serious and committed…” [Participant 8].*

*“…because we do not want them to go back to misuse substances, we teach them some skills such as hair dressing and tv and phone repairs so that they can start to make a living and not become dependent and abuse substances as a form of coping mechanism…” [Participant 4].*

##### **Advocacy and awareness creation**

A major theme that emerged to define another role of mental health oriented NGOs in Ghana is advocacy and awareness creation. Most NGOs have monthly and yearly schedules set aside to lobby and advocate on behalf of their clients. In the current context, NGOs and non-profit organisations are collaborating with other like-minded agencies to advocate for compulsory treatment, instead of incarceration of individuals who attempt suicide. There is evidence that suggests that some individuals with particular types of SUDs may be more likely to have suicidal ideations, attempt suicide, or become violent [27]. In furtherance, NGOs engage with religious and academic institutions to organize forums and symposiums to dispel myths about alcohol and drugs and educate the audience about the dangers of substance misuse. Three subthemes describe these activities: education and sensitization, health/recovery walk, and recovery fair.

### Education and sensitization

In addition to providing treatment and recovery support services for individuals with SUDs, NGOs are also keen at providing education and sensitization on substance misuse prevention among the general populace, and young adults in particular – who are also most vulnerable. NGOs collaborate with school authorities and religious organizations to provide education and sensitization on mental health issues, including substance misuse and its prevention. These discussions are often held in assembly halls of schools, churches, and Mosques and are facilitated by licensed mental health professionals. Discussions range from general expositions on the types of substances commonly misused, through how to identify whether a person may have a SUD, to relapse prevention measures. A participant, a senior recovery practitioner, 41 years, recounted:

*“…we go to speak in schools and churches to enlighten the people about the psychosocial and economic consequences of abusing illicit drugs and how to prevent substance misuse…in one school a student said he was told that smoking marijuana will make him become smarter in school…” [Participant 8].*

*“…we visit ghettos and organizsations like churches and schools to talk about drug and alcohol abuse and its prevention…for the past year, I have been on different TV and radio stations to talk about substance abuse and addiction. Last two weeks Sunday I was on Atinka FM…they also came here to interview some of the clients and myself…I’ve been on Joy Prime… I’ve been on GTV… I’ve been on Metro TV… I’ve been on TV Africa on three occasions…I’ve been on Unique FM…to let people be aware about the drug abuse menace…” [Participant 2].*

### Health/recovery walk

To convey their messages down to the members of the communities where they serve, mental health-oriented NGOs also embark on health walks as part of their awareness creation and sensitization efforts. These walks are often organized annually by the regional associations of NGOs and NPOs in (mental) health and done through the principal streets of Accra. A participant, a 38-year old senior recovery practitioner narrated:

*“…for the recovery walk organized in July each year, we join other NGOs interested in substance abuse treatment to walk with placards and music through the streets as part of sensitization. Last year was in Ashaiman and two years ago was in Accra. We walked from Adabraka cluster of schools through the principal streets of Accra to the Community Centre. We also organize similar activities on the International Day Against Drug Abuse in June every year…” [Participant 7].*

*“…usually we organize programmes to create awareness…and walk through the towns to send down our messages to the people…particularly the youth…about substance abuse and its harmful effects…we also do recovery faires at our facility, where those who have recovered and are doing well in their communities come around to speak and motivate those who are still at the facility receiving treatment…” [Participant 5].*

#### Challenges confronting operationalization

As a secondary objective, we explored the challenges confronting mental health oriented NGOs in their operationalization in Ghana. The data revealed two main challenges, including limited qualified staff and lack of government support.

### Limited qualified professionals

Given that the core functions of these NGOs require the provision of clinical and therapeutic services to clients, the role of mental health professionals are essential to the success of the functions and goals of these NGOs. Yet, presently in Ghana, there is a limited availability of trained and licensed mental health personnel who are knowledgeable in treating and supporting individuals with SUDs. The majority of personnel who work with these NGOs are engaged on part-time basis and attend to clients once-weekly or as necessary. This has significant negative impact on the overall outputs of the NGOs. A director commented:

*“…our main challenge is that it is difficult to come by and hire a clinical psychologist or psychotherapist on full time…there are only a few of them and most of them are already engaged elsewhere. The medical doctors too do not have the required training to treat substance abuse cases…they always refer us to get a psychologist or psychiatrist…” [Participant 3].*

*“…some of our staff are community (mental) health workers with social work background…but they cannot provide psychotherapy and the other services that the clients need…so it is a big problem…ideally we would need a full time psychotherapist or clinical psychologist, but they are not so available and it will be also very expensive to engage them full time since we have limited funds…” [Participant 2].*

#### Inadequate government support

The majority of NGOs are funded by foreign and local donor agencies who often make annual donations. These NGOs thus operate with limited funds and resources to cover their operational cost, which includes salaries and remuneration for full- and part-time staff, rent, and budget for scheduled programs, fuel, and medical care for staff and clients. Participants accentuated that their financial burdens could be lessened by governments’ support by assisting them to have easy access to logistics and facilities, posting mental health staff to work with NGOs, or paying the salaries of trained staff who work with NGOs. A male director, 54-years, mentioned:

*“…we are all trying to help, but our funds are very limited…sometimes churches and few individuals come in, to support…but the government can also assist us to pay the salaries of the personnel who work here…we do not receive any support from the Mental Health Authority…the assemblies sometimes assist us to organize our programs, but they do not give any financial support…” [Participant 1].*

*“…we do not have rehab centers in this country…we are trying with the little we have to run treatment and rehab here…but nobody cares about our challenges…sometimes we run out of money and we have to stop bringing in new clients or discharge some who have made few progress…this is a big challenge…government must help us…” [Participant 8].*

## Discussion

This study sets out to examine the roles of recovery-focused, health-oriented NGOs in the recovery support of persons with SUDs in Ghana, within a qualitative approach. The findings demonstrate that, overall, these NGOs provide three main services in their treatment and recovery management efforts. These include treatment and recovery support services for persons with SUDs using psychotherapy and recovery capital, re-integration of recovered individuals into society, and advocating and creating awareness in schools and communities about the adverse effects of substance misuse. In furtherance, the findings underscore the challenges encountered by NGOs in their operationalization which included limited qualified staff and the lack of government support.

First, findings from the present study suggest that the provision of treatment and recovery support services for individuals with SUDs is one of the main priorities of mental health oriented NGOs in Ghana. With the current model of care, individuals with problem behaviors, including those with SUDs, require a holistic approach of care – which includes treatment for associated psychosocial problems and recovery support [2, 6, 8]. Although governments and health agencies have made some efforts to provide holistic services at the three main psychiatric hospitals in Ghana in the past decades, much of these efforts have focused on the medical treatment component of care, with limited attention to the psycho-social needs of individuals with SUDs or other drug-related problems [28]. Nonetheless, more recently, the Ministry of Health has employed a number of clinical psychologists and psychiatrists at the three main psychiatric hospitals and at all regional hospitals [10, 29].

With the limited number  of trained mental health personnel to cater for the mental health needs of the populace, including individuals with SUDs, many health oriented NGOs have ventured into providing psychological treatment and recovery support services for individuals with SUDs who are under their care [20]. Operating within a holistic, biopsycho-social model, and based on the availability of resources and personnel, the majority of these NGOs have engaged psychologists on part- or full-time basis to provide one or a combination of treatment services, such as psychotherapy and counselling for individuals and groups, cognitive retraining, family support and counselling, and vocational and livelihood skills training. Prior to their admission to the facilities, in-coming individuals with SUDs are often taken to a psychiatrist for psychiatric assessment. When necessary, the individual is given some psychotropic medications to treat particular symptoms. The NGOs also occasionally invite a psychiatrist to their facilities or take clients who exhibit severe withdrawal symptoms to an associated psychiatrist or psychologist for attention.

In addition to providing treatment and recovery support services for their clients, we also observed that the majority of the NGOs in the present study also organize vocational training in electronic repairs (e.g., television, sound systems, and radio sets), carpentry, and printing for clients. Tailored specifically for individuals without a skilled job, artisans with the requisite training and skills are invited to train a selected group of clients at their facilities on a weekly basis. These trainings are also attended by non-residential, previously discharged individuals. After the completion of the training, the participants are given start-up capitals and some resources to enable them to start work when they are discharged from the facilities. The provision of these trainings and subsequent support serve as important preventive strategy, considering that boredom, lack of employment, and poverty have previously been identified as factors that precipitate substance misuse and relapse to substance misuse in Ghana [28].

While social support has been widely considered as a critical component of the SUDs treatment and recovery process by facilitating home and job re-integration process [17, 20], there has been a lack of support by some family members for relatives who have recovered from SUDs or mental illness in general [13, 30]. Previous research has shown that most family members do not visit or participate in the recovery process of relatives receiving mental health treatment at the psychiatric hospitals [30]. Studies and media reports also indicate that some families do not welcome or support relatives who have been treatment and discharged from psychiatric hospitals back into their homes [31]. These actions by some families may be fuelled by a combination of cultural and social factors, as well as misinformation or low level knowledge about mental health. Particularly in the non-urban context of Ghana, a proportion of the population still considers  all forms of mental health problems as shameful, transmittable, and caused by evil spirits [31, 32]. To this extent, some family members explicitly disassociate themselves with relatives who had some form of mental health problems and may be unwilling to take them back and offer them the needed social support to facilitate their recovery. To counteract this phenomenon, NGOs have instituted approaches to educate communities and family members on mental health and the important roles they can play to facilitate the recovery of their family members with mental health problems, including those with SUDs. These mental health oriented NGOs collaborate with other NGOs, governmental health institutions, and other agencies (e.g., Social Welfare) to educate, support, or compel families and individuals responsible for recovered individuals to support them during the treatment process and upon discharge from the facilities.

The findings of the current study showed that NGOs collaborate with the Department of Social Welfare and related agencies to facilitate the re-integration of recovered individuals back to their previous jobs. While this process is often seamless for clients who worked in the civil service and other government sectors, the process is often challenging and usually require legal actions in the case of individuals from private organizations – who often do not have valid work contracts. In such instances, the NGOs rely on the national labour and employment laws of Ghana to compel reluctant organisations to re-engage these recovered individuals. In cases where the nature of the job could have played a role in the misuse of substances (e.g., individuals who work at alcoholic distilleries or involved in very stressful, manual jobs), it is explained to the organization involved the possibility that the stressful and physically demanding nature of the job could contribute to the worker's misuse of illicit psychoactive substances. When necessary, the NGOs support the individual in court to compel the organization to re-engage or compensate the client.

Another major role of mental health oriented NGOs in Ghana is the provision of education on substance misuse and advocating for the needs of individuals with SUDs and other forms of mental illness. Presently, there is limited awareness of the considerable advances in the knowledge of the causes and treatment of SUDs and mental illness, more generally, in Ghana. Recent studies have nonetheless explored the precipitants of substance misuse in Ghana [28], relapse prevention strategies utilised by individuals one year and four after treatment for poly-substance use disorder at a psychiatric unit in Ghana [14, 33]. Collaborating with organizations, government agencies, communities, and community leaders, mental health oriented NGOs have embarked on awareness creation in schools, churches, and communities to provide factual information on the prevalence, precipitants, harmful and psycho-social effects of substance misuse, as well as on the existing treatment and recovery support options available for individuals with SUDs and other illicit drugs related problem behaviors. In their recent engagements, four of the participating NGOs, for instance, embarked on media campaigns to lobby policy makers to allocate significant proportion of the annual health budget to mental health in order to improve mental healthcare in general. Generally, these NGOs have communicated their messages to students and community members, through films and drama, in the metropolis within which they operate. For instance, films on stigma, discrimination against individuals with mental health problems, and the need to provide support for this vulnerable group have been compiled and shown in schools and at community centres.

A few challenges confront these NGOs in their quest to provide these services to clients. First, considering the specialized functions and roles of these NGOs, there is need to engage well-trained mental health personnel with the requisite skills for the various roles that these NGOs engage in. However, NGOs are faced with the challenge of competing with private organisations and government institutions to hire clinical psychologists and psychiatrists – who are presently very few in Ghana. A total of 25 psychologists were recruited by the Ministry of Health in 2013 [28], with a dozen more employed by Universities and private health institutions. For this reason, a few NGOs have sponsored promising staff with the requisite educational backgrounds to pursue postgraduate studies in mental health to fill their needs.

Second, from the analysis, NGOs also report of inadequate government support and interventions to lessen the financial constraints. Some NGOs expect, for instance, that government support them to pay (or share) the salaries of mental health and other professionals who are employed on full-time in their facilities. Some group of professionals, such as clinical psychologists, with advanced training (e.g., masters and PhD levels) require  high corresponding salary and benefits – which are often beyond the budgets of these NGOs. They also expect some tax holidays on their equipment and logistics that they purchase or import into the country. Third, psychotropic medications for the treatment of SUDs, and mental disorders more generally, are supplied by government to the public psychiatric facilities and administered to clients at no cost [34]. However, there are occasional acute shortages of psychotropic medications that thwart the efforts of the NGOs in the recovery support services for their clients [24]. In such instances, NGOs resort to buying from pharmaceutical companies, which can be expensive.

Although this study contributes to the literature, as the first, to our knowledge, to provide in-depth information about the roles and contributions of NGOs in the recovery support for individuals with SUDs in Ghana, we acknowledge the following limitations. First, all participants in this study were personnel working at health-based NGOs. It is possible that some participants may have provided socially desirable responses or overemphasize the role and contributions of their organizations in the recovery management of individuals with SUDs. Future studies should triangulate to include views from recovered or discharged clients and their families. Second, this qualitative study recruited a small sample of eight participants. Although data saturation was reached, caution should be applied in interpreting, inferring, and generalizing the findings, due to the small sample size. We recommend that future studies adopt a mixed method approach to recruit a larger sample from various NGOs across the country. Third, the study only focused on NGOs that provide mental health care and SUD recovery support services to clients. It is possible that other non-mental health NGOs may also provide direct or indirect services to support clients with SUDs. It may be useful for future studies to also explore the extent to which other non-mental health NGOs are involved in the recovery support services.

## Conclusions

NGOs involved in (mental) healthcare in Ghana, have made remarkable contributions by complementing the efforts by state institutions in the provision of treatment and recovery support services for individuals with SUDs, in the midst of considerable challenges with logistics and shortage of qualified personnel. While the contributions of health-oriented NGOs in Ghana have not been disputed, this study is the first, to our knowledge, to examine the roles they play in recovery process for individuals with SUDs. In many ways the models and strategies adopted by these NGOs can serve as useful guide for the public healthcare system to emulate. The operational challenges faced by these NGOs have significant impact on their efforts, which prevent them from meeting their targets of providing care for all individuals with SUDs in need of care. There is need for mental health related government agencies to support the roles of these NGOs and form mutually-beneficial collaborations to enhance and advance their agenda and efforts.

## Data Availability

Data is available upon reasonable request from the first and corresponding author.

## Abbreviations

GAR: Greater Accra Region
NGOs: Non-Governmental Organisations
NPOs: Non-profit organizations
SUDs: Substance Use Disorders
UNODC: United Nations Office on Drugs and Crime
